# Effect of canalith repositioning on resting-state brain functional connectivity in patients with benign paroxysmal positional vertigo

**DOI:** 10.3389/fneur.2025.1681447

**Published:** 2026-01-21

**Authors:** Wenjia He, Xinyu Lyu, Hui Zhang, Meng Wang, Lihong Zhai, Zhanguo Jin

**Affiliations:** 1 School of Public Health, North China University of Science and Technology, Tangshan, China; 2 Research Center for Air and Space Medicine and Vertigo Diagnosis and Treatment, Air Force General Hospital PLA, Beijing, China

**Keywords:** benign paroxysmal positional vertigo, functional near-infrared spectroscopy, canalith repositioning maneuver, resting-state, functional connectivity

## Abstract

**Objective:**

To compare the characteristics of resting-state functional connectivity (FC) before and after repositioning therapy in patients with benign paroxysmal positional vertigo (BPPV) using functional near-infrared spectroscopy (fNIRS).

**Methods:**

Fifty BPPV patients and fifty healthy controls were enrolled. Oxygenated hemoglobin (HbO) concentration changes during resting-state were recorded using fNIRS. The experimental group underwent Dizziness Handicap Inventory (DHI), Visual Analogue Scale (VAS) assessments, and 10-min resting-state fNIRS scans before and after repositioning therapy; the control group received baseline scans only. FC strength of the whole brain and specific regions of interest (ROIs) was analyzed using correlation coefficients.

**Results:**

fNIRS analysis revealed significantly elevated FC strength between the middle temporal gyrus (MTG) and both the motor cortex (MC) and somatosensory cortex (SC) in BPPV patients at baseline compared to healthy controls (*p* < 0.05); after canalith repositioning, the whole-brain average FC strength in patients significantly decreased. Connectivity strength decreased synchronously in the following ROI pairs: prefrontal cortex (PFC)-occipital cortex (OC), PFC-MTG, PFC-MC, OC-MC, OC-SC, MTG-MC, and MTG-SC, and brain network parameters returned to normal levels post-repositioning. Clinical indicators improved simultaneously: the total DHI score decreased by 23.4% (*p* < 0.05), and the VAS score showed a significant reduction.

**Conclusion:**

BPPV involves compensatory enhancement of the vestibulo-sensorimotor network. Canalith repositioning eliminates abnormal vestibular input and restores pathologically enhanced FC to normal levels. This supports fNIRS as a potential objective neuroimaging biomarker for evaluating BPPV neural mechanisms and treatment efficacy.

## 1 Introduction

Benign paroxysmal positional vertigo (BPPV) is one of the most common peripheral vestibular disorders causing vertigo, accounting for 17–42% of all vertigo patients (1). It is characterized by transient vertigo and characteristic nystagmus induced by specific head position changes, with symptoms typically lasting no longer than 60 s (2, 3). BPPV presents diverse clinical manifestations including imbalance, nausea, and vomiting, and is more prevalent among elderly populations (4, 5). The currently accepted pathogenesis involves abnormal detachment of calcium carbonate crystals from the utricular macula that migrate into the semicircular canals. These dislodged otoconia may enhance vestibular sensitivity to gravitational stimuli, thereby triggering vertigo and nystagmus (6, 7). Additionally, BPPV development associates with multiple risk factors including age, sex, head trauma, hypertension, migraine, and hyperlipidemia (8). Functional near-infrared spectroscopy (fNIRS) is a non-invasive brain monitoring technique based on hemodynamic responses. fNIRS relies the penetrability of near-infrared light (650–950 nm) through biological tissues and differential light absorption properties of hemoglobin species (oxygenated HbO, deoxygenated HbR) (9, 10). By applying the modified Beer–Lambert law to quantify light attenuation between emitter and detector, it calculates relative changes in cortical hemoglobin concentrations (11). fNIRS offers advantages of non-invasiveness, portability, and low cost, making it suitable for diverse brain function studies including cognition, emotion, and motor control (12, 13). fNIRS applications in neuroscience and clinical medicine continue to expand, particularly in investigating neural response mechanisms to specific stimuli (14). fNIRS demonstrates unique strengths in studying neurodevelopmental mechanisms and disease pathology in infants and special populations, providing critical insights (15). Furthermore, it enables assessment of therapeutic interventions on cerebral function, offering objective evidence for treatment efficacy evaluation and personalized regimen optimization (16). Its tolerance to motion artifacts makes it particularly suitable for studying brain activity during naturalistic contexts such as walking, social interaction, and rehabilitation training (17).

This study aims to utilize fNIRS technology for analyzing FC strength of HbO signals during resting-state before and after repositioning therapy, thereby investigating its effects on resting-state functional brain networks in BPPV patients. This approach will elucidate the neural mechanisms underlying therapeutic effects, advance understanding of BPPV pathophysiology and post-treatment neuroplastic reorganization, and potentially provide novel neuroimaging biomarkers for evaluating repositioning efficacy and optimizing therapeutic strategies-ultimately contributing to precision diagnostics and treatment of BPPV.

## 2 Materials and methods

### 2.1 Participants

Fifty patients with benign paroxysmal positional vertigo (BPPV) admitted to the Vertigo Center of the Air Force Medical Center (July 2024–July 2025) were enrolled as the experimental group (17 males, 33 females; mean age 49.50 ± 11.64 years), comprising 38 posterior semicircular canal canalithiasis (PSC-Can) and 12 horizontal semicircular canal canalithiasis (HSC-Can) cases. Inclusion criteria (18–21): (a) diagnosis of primary unilateral PSC-Can or HSC-Can confirmed by medical history and characteristic nystagmus during Dix-Hallpike and roll tests; (b) successful canalith repositioning with resolution of characteristic nystagmus and negative positional testing. Exclusion criteria (22): (a) specific BPPV subtypes (bilateral BPPV, ipsilateral multi-canal BPPV, or light cupula); (b) other peripheral vestibular or central neurological vertigo; (c) use of medications affecting central nervous or vestibular system function within 48 h prior to assessment; (d) history of head trauma or otologic surgery within 6 months. Fifty healthy volunteers (19 males, 31 females, mean age 46.30 ± 13.25 years) recruited concurrently served as controls. Inclusion criteria: (a) no history of vertigo or balance disorders; (b) absence of neurological/psychiatric conditions. Exclusion criteria: (a) current vestibular or neurological disorders; (b) inability to comply with testing; (c) use of medications affecting central nervous or vestibular system function within 48 h prior to assessment; (d) history of head trauma or otologic surgery within 6 months. The protocol was approved by the Ethics Committee of the Air Force Medical Center (Approval No.: 2024-91-PJ01), with all participants providing written informed consent after full disclosure of study objectives, procedures, and potential risks.

### 2.2 Experimental equipment and scales

This study employed the VertiChair BPPV diagnostic and treatment system for both diagnosis and canalith repositioning therapy. In cases of canalithiasis of the posterior semicircular canal, the Dix-Hallpike test was used for diagnosis and the Epley maneuver was employed for repositioning. For canalithiasis of the horizontal semicircular canal, the Roll test was used for diagnosis, and either the Barbecue or the Gufoni maneuver was utilized for repositioning. The NirSmart-500pro device was used to acquire resting-state data from BPPV patients. Clinical symptom assessment included the Dizziness Handicap Inventory (DHI) to quantify the impact of the disease on daily living activities, and the Visual Analogue Scale (VAS) was applied to evaluate the subjective severity of vertigo symptoms.

### 2.3 Data acquisition and Preprocessing

**Figure 1:**
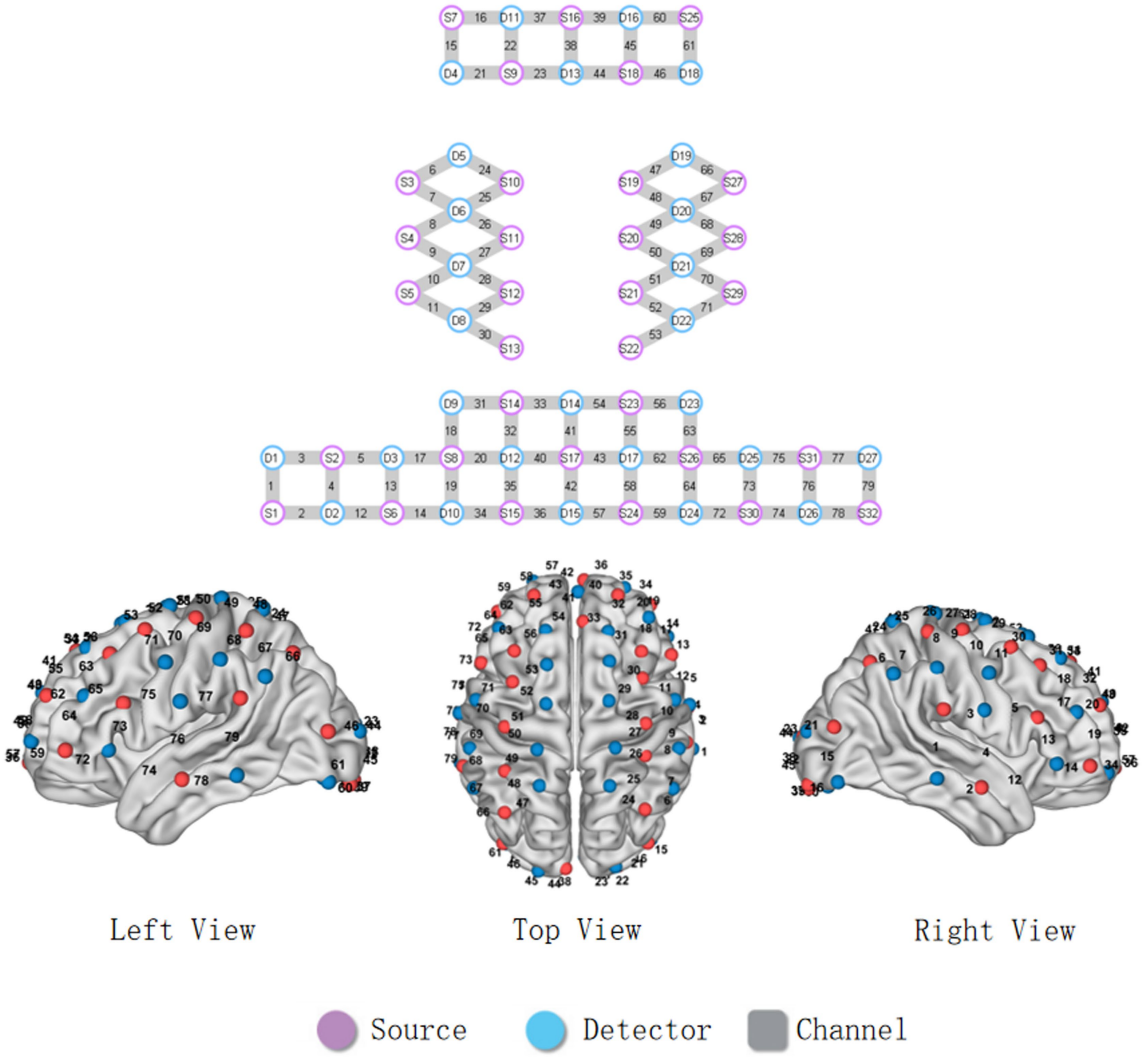
Configuration of fNIRS channels. The red dots represent the light sources, and the blue dots represent the light detectors. In total, 32 sources and 27 detectors resulted in 79 channels encompassing five regions of interest, specifically bilateral prefrontal cortex (PFC), middle temporal gyrus (MTG), bilateral motor cortex (MC), and bilateral somatosensory cortex (SC).

This experiment employed the NirSmart-500pro functional near-infrared spectroscopy system (Danyang Huichuang Medical Equipment Co., Ltd., Jiangsu, China) to continuously measure and record concentration changes of HbO and deoxygenated hemoglobin (HbR) in participants’ brains, utilizing light-emitting diodes (LEDs) as light sources and avalanche photodiodes (APDs) as detectors, with emitter probes delivering near-infrared light at 730 nm and 850 nm wavelengths at a sampling rate of 10 Hz, using a cap equipped with 32 light sources and 27 detectors configured to form 79 effective measurement channels, where the average source-detector separation was 3 cm and the fNIRS acquisition cap channel schematic is detailed in Figure 1. Based on previous studies (23, 24), the measurement channels were classified into five ROIs: prefrontal cortex (PFC), middle temporal gyrus (MTG), bilateral motor cortex (MC), and bilateral somatosensory cortex (SC). Both oxygenated (HbO) and deoxygenated hemoglobin (HbR) concentrations were recorded. However, due to its higher signal-to-noise ratio in functional connectivity studies, subsequent analyses focused solely on the HbO signal (25–27).

Participants first completed the DHI and VAS scales. Subsequently. Prior to resting-state data collection, participants rested in a comfortable seated position for 3 min to achieve a calm state before donning the fNIRS cap; during formal acquisition, subjects were instructed to maintain physical stillness and relaxation while avoiding deliberate thinking or counting, with eyes focused on a central “+” fixation cross displayed on a front-positioned screen, during which 10 min of resting-state fNIRS data were continuously acquired. BPPV patients who successfully underwent canalith repositioning treatment were scheduled for a second fNIRS data acquisition 30 min post-treatment, during which they then completed the DHI and VAS scales a second time.

This study utilized the NirSpark software package (V1.8.9, Huichuang, China) to perform standardized preprocessing of the fNIRS signals. The procedure was conducted as follows (28, 29): First, raw data underwent quality inspection. Channels with signal intensity below 5,000 μW/cm^2^ were automatically flagged as saturated and excluded. Motion-contaminated segments, identified by a standard deviation exceeding 0.5 μM within a 0.5-s window, were also automatically marked for rejection. This automatic screening was followed by manual review to further ensure data quality. Subsequently, motion artifacts were corrected on a channel-wise basis using a spline interpolation algorithm (*λ* = 6, cubic spline), an effective method for eliminating pulse-like spikes or signal dropouts caused by probe displacement. A band-pass filter of 0.01–0.08 Hz was then applied to attenuate physiological noises, such as respiration (0.2–0.3 Hz) and heart rate (0.8–1.2 Hz), while preserving the frequency band characteristic of hemodynamic responses. Finally, the optical density signals were converted into concentration changes of HbO based on the modified Beer–Lambert law. FC strength between channels was quantified by calculating Pearson’s correlation coefficients. These correlation coefficients subsequently underwent Fisher’s *Z*-transformation to normalize their distribution for further statistical analysis.

### 2.4 Sample size calculation

As there is currently no published literature on FC values in patients with benign paroxysmal positional vertigo, the sample size for this study was determined primarily based on a preliminary pilot study. According to the pre-experiment data, the standard deviation of the paired differences (σd) was estimated to be 0.1, and the mean difference (*δ*) was 0.048. The significance level (*α*) was set at 0.05 (two-tailed), and the statistical power (1 − *β*) was set at 0.90. Using the sample size formula for paired designs:


$$n={\left[\frac{(Z_{1-\alpha /2}+Z_{1-\beta })\sigma_{d}}{\delta }\right]}^{2}$$

The calculated minimum required sample size was 45 subjects. To enhance the reliability of the study and account for a potential 10% rate of loss to follow-up or data exclusion, the final sample size was increased to 50 subjects.

### 2.5 Statistical analysis

Intergroup differences between healthy controls and benign paroxysmal positional vertigo (BPPV) patients were assessed using independent samples *t*-tests for resting-state FC values and age, while χ^2^ tests compared gender, education level, and the prevalence of underlying conditions such as hypertension, diabetes, and hyperlipidemia; paired samples *t*-tests analyzed pre- versus post-repositioning resting-state FC values and scale scores in the BPPV group, with statistical significance set at *p* < 0.05. Additionally, Spearman correlation analysis was performed to assess the relationships between the DHI scores, VAS scores, and the whole-brain mean FC strength, as well as the relationship between VAS scores and visual cortex regions.

## 3 Results

### 3.1 Baseline characteristics of study participants

No statistically significant differences were observed between the two groups in terms of gender, age, or education level (*p* > 0.05) (Table 1).

**Table 1:** Comparison of baseline levels between the two groups of subjects.

Variable	BPPV (*n* = 50)	HC (*n* = 50)	t/χ^2^	*p*
Age (years) (x̄ ± s)	49.50 ± 11.64	46.30 ± 13.25	1.283	0.203
Gender [*n* (%)]			0.174	0.677
Male	17	19		
Female	33	31		
Educational level [*n* (%)]			0.809	0.667
Junior high school, primary school or below	5	5		
Secondary school / High school	5	8		
College / University or above	40	37		
With underlying diseases [*n* (%)]				
Hypertension	22	18	0.571	0.750
Hyperlipidemia	16	14	0.148	0.700
Diabetes Mellitus	18	16	0.148	0.700

### 3.2 fNIRS resting-state functional connectivity results

#### 3.2.1 Baseline differences

The demographic and clinical baseline characteristics of the BPPV patients and healthy controls are summarized in Table 1 and Table 2, respectively.

**Table 2:** Clinical characteristics of patients with BPPV.

Clinical features	BPPV (*n* = 50)
Affected semicircular canals	
Posterior semicircular canal	38 (76%)
Horizontal semicircular canal	12 (24%)
Affected side	
Left side	17 (34%)
Right side	33 (66%)
Disease course	
< 3 days	25 (50%)
3 days to 3 weeks	20 (40%)
3 weeks to 3 months	5 (10%)
Duration of vertigo (weeks)	
< 1	22 (44%)
≥1	28 (56%)
Episode frequency (daily frequency)	
1–2	18 (36%)
≥3	32 (64%)
Number of repositioning maneuvers (times)	
1–2	27 (54%)
≥3	23 (46%)

Baseline resting-state FC analysis revealed multilevel differences between BPPV and control groups:

At the whole-brain level, no statistically significant difference existed in average FC strength (*t* = 1.695, *p* > 0.05).

At the ROIs level, significantly higher FC strength was observed between specific regional pairs in BPPV patients, specifically showing enhanced connectivity between the MTG-MC (*t* = 3.341, *p* < 0.01), as well as between MTG-SC (*t* = 3.645, *p* < 0.01).

#### 3.2.2 Repositioning effects

**Figure 2:**
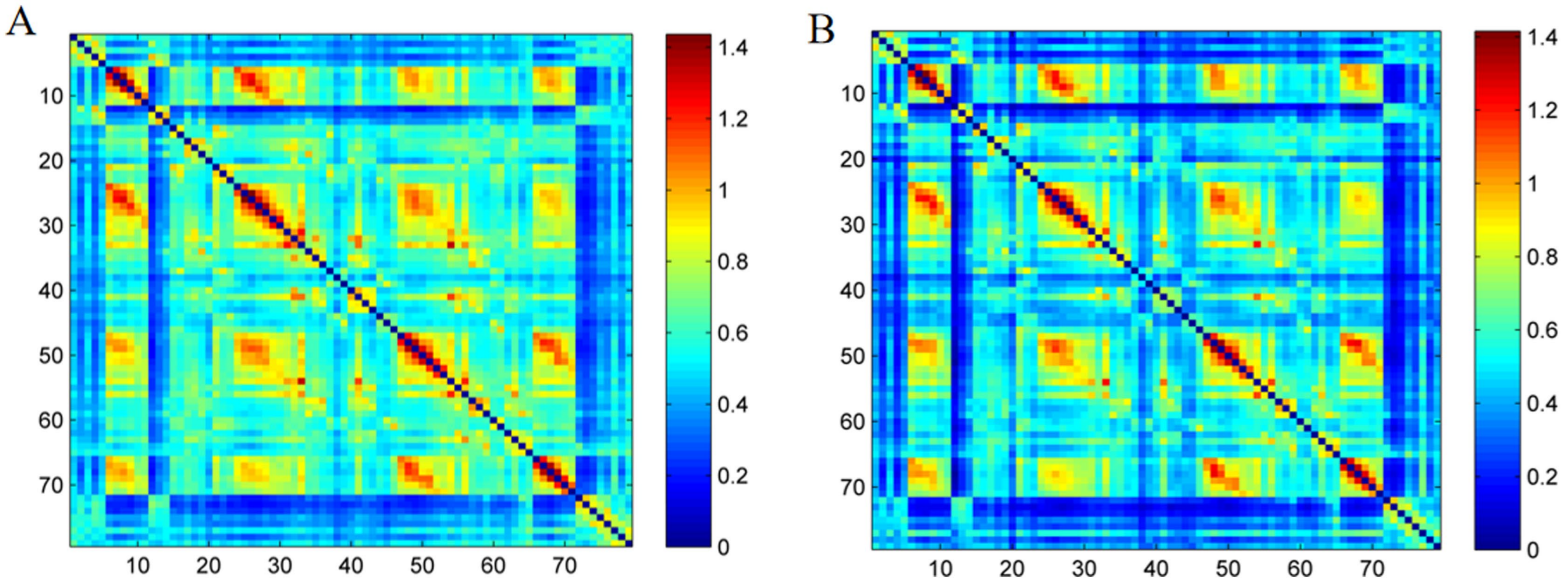
Group-averaged resting-state functional connectivity (RSFC) matrix diagram. **(A)** RSFC matrix of the BPPV group before repositioning. **(B)** RSFC matrix of the BPPV group after repositioning.

Canalith repositioning significantly altered resting-state FC patterns in BPPV patients:

Whole-brain analysis demonstrated significantly higher pre-repositioning average FC strength (0.58 ± 0.22) versus post-repositioning (0.50 ± 0.20) (*t* = 3.171, *p* < 0.01). The group-averaged FC for each group is shown in Figure 2.

**Figure 3:**
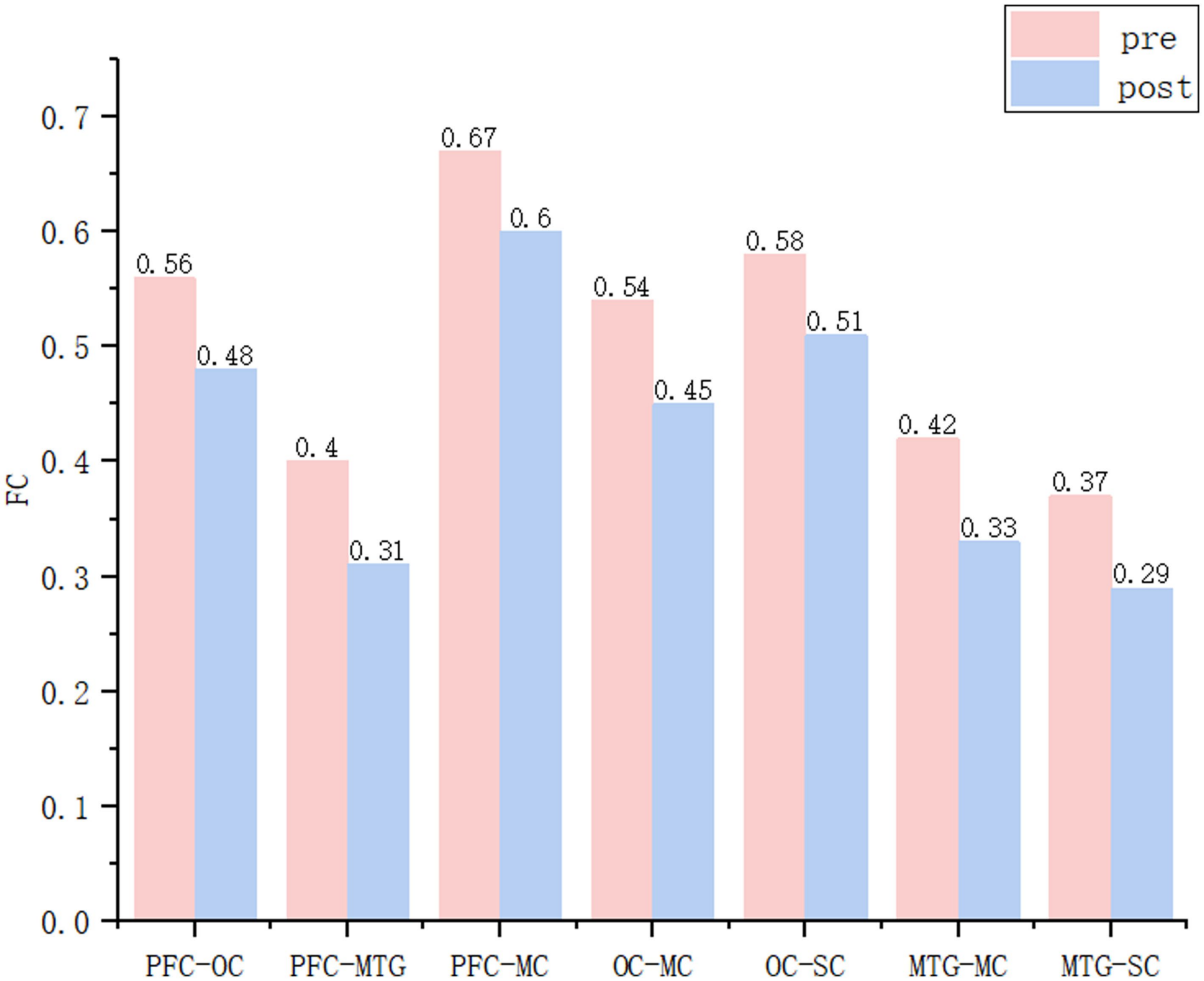
BPPV group significant difference in ROIs connectivity before and after repositioning figure. PFC, prefrontal cortex; MTG, middle temporal gyrus; MC, motor cortex; SC, somatosensory cortex; OC, occipital cortex.

ROI-level analysis revealed significantly reduced FC strength after repositioning in seven region pairs. These included PFC-OC (*t* = 2.832, *p* < 0.05), PFC-MTG (*t* = 2.713, *p* < 0.05), PFC-MC (*t* = 2.192, *p* < 0.05), OC-MC (*t* = 3.235, *p* < 0.01), OC-SC (*t* = 3.088, *p* < 0.01), MTG-MC (*t* = 3.461, *p* < 0.01), and MTG-SC (*t* = 3.034, *p* < 0.01) (Figure 3).

#### 3.2.3 Post-repositioning status

At both the whole-brain level and in specific ROIs, no statistically significant differences in mean FC strength were observed between the patient group and the control group (*p* > 0.05).

### 3.3 Scale assessments

Correlation analysis demonstrated positive correlations between Dizziness Handicap Inventory (DHI)/Visual Analogue Scale (VAS) scores and whole-brain mean FC strength, where higher FC values corresponded to elevated scale scores (DHI: *rs* = 0.626, *p* < 0.01; VAS: *r*s = 0.674, *p* < 0.01).

To explore the neural mechanisms underlying visual vertigo, we further investigated the correlation between the VAS scores and FC strength involving the OC before and after repositioning. Spearman correlation analysis revealed that pre-repositioning VAS scores were significantly positively correlated with the strength of FC in the PFC-OC (*rs = 0.576*, *p* < 0.01), OC-MC (*rs = 0.548*, *p* < 0.01), and OC-SC (*rs* = 0.428, *p* < 0.01) pathways. However, following successful repositioning, these correlations were no longer statistically significant (*p* > 0.05).

**Figure 4:**
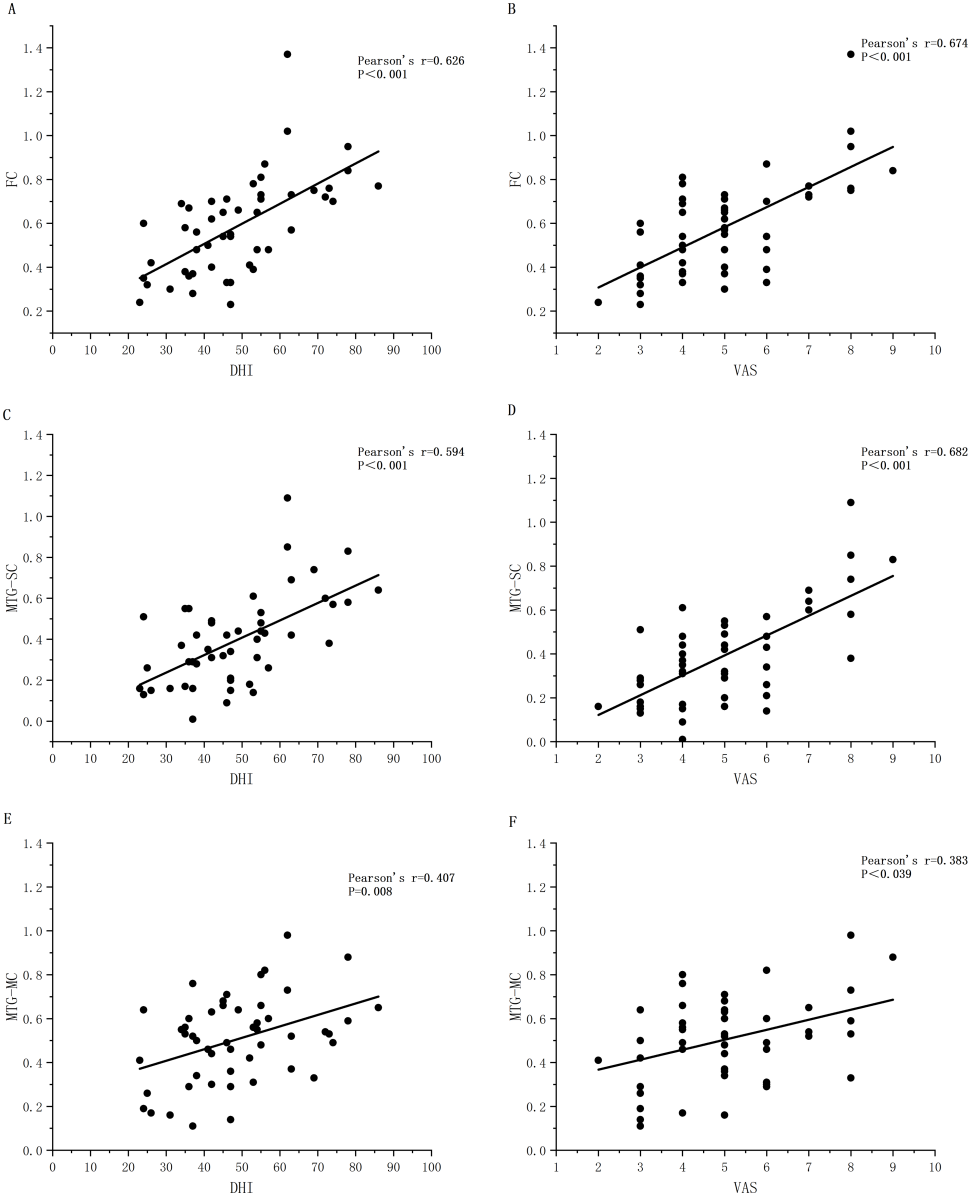
Scatter plots of Spearman correlation analysis between FC of whole-brain and different ROIs and DHI scores as well as VAS scores. PFC, prefrontal cortex; MC, motor cortex; SC, somatosensory cortex. The ‘rs’ is the correlation coefficient of Spearman’s analysis. **(A)** Significant correlation between DHI and whole-brain FC. **(B)** Significant correlation between VAS and whole-brain FC. **(C)** Significant correlation between DHI and FC of SC. **(D)** Significant correlation between VAS and FC of SC. **(E)** Significant correlation between DHI and FC of MC. **(F)** Significant correlation between VAS and FC of MC.

Concurrently, DHI and VAS scores exhibited positive correlations with MTG-MC and MTG-SC connectivity strength within ROIs, with increased connectivity values associating with higher scale scores, as illustrated in Figure 4.

The BPPV cohort completed scale evaluations both before and after repositioning. Paired *t*-tests revealed significantly reduced DHI total scores after repositioning (pre: 38.46 ± 19.15; post: 29.44 ± 14.70; *t* = 3.894, *p* < 0.01), indicating a mean decrease of 9.02 points (a 23.4% reduction). VAS scores were also significantly reduced (pre: 4.56 ± 1.62; post: 3.40 ± 2.10; *t* = 3.732, *p* < 0.01).

## 4 Discussion

### 4.1 Elevated pre-treatment connectivity and compensatory reorganization

This study utilized fNIRS to investigate resting-state FC characteristics in BPPV patients versus healthy controls before and after repositioning. Results demonstrated significantly elevated pre-repositioning mean connectivity strength in BPPV patients compared to controls, with marked reduction post-repositioning, providing insights into the neuromodulatory mechanisms of canalith repositioning. Critically, we observed enhanced FC between MTG and MC, as well as MTG and SC in BPPV patients before treatment, indicating compensatory reorganization of brain functional networks.

The core pathology of BPPV involves otoconial detachment from the utricular/saccular maculae causing cupular displacement in semicircular canals, leading to abnormal vestibulo-ocular reflexes and postural imbalance (30). This aberrant vestibular input persistently challenges brain networks responsible for spatial orientation, balance perception, and multisensory integration (31). The MTG—particularly near the temporoparietal junction—serves as a key cortical hub for vestibular processing and spatial integration, receiving ascending projections from vestibular nuclei to construct head position and motion awareness (32, 33). The MC plans and executes motor commands crucial for postural control (34), while the SC processes proprioceptive and tactile feedback essential for maintaining static/dynamic equilibrium (35, 36). As documented in the literature, patients with bilateral vestibular loss fail to achieve ‘sensory channel reorganization,’ resulting in impaired balance across varying environmental conditions. This underscores the vestibular system’s pivotal role in multisensory integration, with its dysfunction severely impairing spatial perception and motor control (37).

Confronted with aberrant vestibular inputs that provoke spatial disorientation and vertigo, the brain activates compensatory mechanisms to preserve postural equilibrium and motor coordination (38). The enhanced MTG-MC connectivity likely reflects intensified coordination wherein the MTG, processing anomalous spatial signals, more robustly directs the MC to execute balance-correcting maneuvers. Stronger FC may signify amplified signaling from MTG to MC regarding spatial errors or imbalance risks, thereby driving more frequent/precise postural adjustments from the MC to counteract vestibular disruption—representing compensatory upregulation of vestibulo-motor pathways.

When unstable spatial orientation arises from pathological vestibular inputs, the brain increases reliance on reliable proprioceptive feedback to calibrate and stabilize spatial perception. One study revealed that posterior canal BPPV patients exhibit greater body sway velocity than healthy individuals during eyes-closed standing (39), indicating heightened dependence on SC-mediated proprioception for spatial calibration in BPPV pathology. Augmented MTG-SC connectivity may embody this increased somatosensory reliance, enabling the MTG to integrate proprioceptive signals from the SC more effectively to “correct” or “counteract” erroneous vestibular inputs, thereby sustaining an accurate spatial body map.

The concurrent strengthening of MTG-MC and MTG-SC FC collectively constitutes a vestibulo-sensorimotor compensatory network. Through enhanced information flow and synergy among key regions responsible for spatial awareness, somatosensory feedback, and motor output, BPPV patients attempt to counterbalance vestibular dysfunction and vertigo-a dynamic neural reorganization exemplifying neuroplastic adaptation to vestibular pathology.

### 4.2 Post-repositioning FC reduction and neural plasticity

The post-repositioning reduction in whole-brain and regional FC strength represents neural plasticity-driven adaptation following normalization of vestibular afferent signals. Mechanistically, successful repositioning restores dislodged otoconia to the utricle, eliminating aberrant endolymph flow dynamics. Consequently, vestibular nerve inputs transition from pathological signaling to physiological feedback, where endolymph movement reflects true angular acceleration rather than erroneous otolith-induced displacements (40). Analogous to stroke rehabilitation where motor cortex connectivity decreases with functional recovery (41), our findings suggest diminished compensatory demands permit downregulation of redundant connections in patients with BPPV. As physiological vestibular inputs resume, the CNS ceases sustained processing of conflicting sensory signals, obviating the need for compensatory neural activation. This adjustment relies on dynamic neuroplasticity: synaptic strength remodeling initially reinforced redundant connectivity during pathology, but post-repositioning, synaptic inhibition reduces synchrony in these pathways, reverting neural circuits to precision collaboration mode—reducing metabolic costs and aberrant signal interference, ultimately manifesting as rationalized FC reduction.

Notably, the observed reduction in FC strength particularly involved the PFC and several other brain regions, such as the OC, MTG, and MC. As a core hub for higher-order cognitive control, multisensory integration, and attentional regulation (42), the enhanced FC strength of the PFC during the pathological state of BPPV and its normalization following repositioning underscore the crucial role of cognitive processes in cerebral compensatory and recovery mechanisms.

During the acute phase of BPPV, persistent conflicts between aberrant vestibular inputs and normal signals from the visual and proprioceptive systems result in spatial disorientation and vertigo. We speculate that, to counteract this conflict and maintain postural stability, the brain not only augments lower-level integration within sensorimotor regions but also recruits higher-order cognitive resources to suppress inaccurate sensory signals, reallocate attention, and execute more sophisticated balance control strategies. The strengthened connectivity between the PFC and the MC, OC, and MTG may reflect such a “cognitive compensation” mechanism. Specifically, enhanced PFC-MC connectivity may indicate increased cognitive supervision for motor planning and execution; heightened PFC-OC connectivity could suggest greater reliance on visual cues and enhanced visuospatial attention to compensate for unreliable vestibular signals; and increased PFC-MTG connectivity might reflect direct PFC modulation of vestibular information processing and conflict resolution.

Successful canalith repositioning eliminates the source of abnormal vestibular input, thereby restoring coordination among the vestibular, visual, and proprioceptive systems. With the resolution of sensory conflict, the brain no longer requires the energy-intensive, PFC-dependent cognitive compensation mechanism. Consequently, the strengthened functional connections between the PFC and sensorimotor/visual regions during the pathological phase are reduced, signifying a return from a conscious, effort-driven control mode to an automated and efficient postural control mode.

This downregulation of PFC-related connectivity, consistent with the overall decrease in global connection strength, represents an important mechanism by which the central nervous system disengages unnecessary compensatory processes, thereby optimizing functional efficiency and conserving energy. It further supports that the neuroplastic changes induced by repositioning therapy extend beyond the sensorimotor level to encompass higher cognitive domains.

In summary, the alterations in PFC connectivity observed in this study indicate that the neural compensation in BPPV involves a multi-level network reorganization ranging from lower-order sensory integration to higher-order cognitive control. Conversely, recovery after repositioning manifests as a synchronous normalization across these hierarchical networks, wherein reduced PFC connectivity serves as an indispensable component, marking the alleviation of cognitive load and the restoration of neural efficiency.

### 4.3 Visual dependence compensation and symptom correlation

This study observed a synchronous reduction in FC between the OC and multiple brain regions, including the PFC, MC, and SC, following repositioning, suggesting significant alterations in neural activity within visual processing pathways after successful treatment. Coupled with the symptomatic improvement assessed by the VAS, we speculate that this phenomenon may be closely related to the compensatory mechanism of visual dependence commonly seen in BPPV and its subsequent resolution (43).

During the acute phase of BPPV, abnormal vestibular input disrupts the balance of multisensory integration. To maintain spatial orientation and postural stability, patients increasingly rely on visual cues. This enhanced visual dependence serves not only as a crucial central compensatory strategy but may also form the neural basis for visual vertigo symptoms. According to the theory of multisensory integration, the brain redistributes sensory weights and increases its reliance on visual signals when vestibular function is compromised (44). Previous studies have indicated that visually dependent individuals exhibit enhanced visual cortex modulation and more pronounced vertigo symptoms when confronted with sensory conflict (45). Therefore, the enhanced connectivity observed in this study between PFC-OC, OC-MC, and OC-SC can be interpreted as a neural representation of cross-modal compensation: the PFC may be involved in the cognitive regulation of visual information processing, while the enhanced coordination between visual, motor, and somatosensory cortices facilitates more precise movement adjustments based on visual cues in dynamic environments.

To directly validate the relationship between visual dependence and clinical symptoms, we further analyzed the correlation between VAS scores and OC-related FC strength. Prior to repositioning, VAS scores showed significant positive correlations with the strength of PFC-OC, OC-MC, and OC-SC connectivity, providing direct evidence for the above hypothesis: the more severe the visual vertigo, the stronger the synergistic activity between the visual network and other brain regions. Following successful repositioning and the elimination of aberrant vestibular afferent signals, these correlations were no longer significant. This indicates that once the abnormal input is removed, the brain’s demand for excessive visual compensation decreases, and the association between FC strength of the visual network and the subjective experience of vertigo is dissociated. Neural activity thus returns from a compensatory, high-load state to an efficient and automated state of balanced multisensory integration.

In summary, this study not only provides objective evidence, for the first time via fNIRS, for the existence and reversibility of visual dependence in BPPV from the perspective of changes in FC strength, but also, through symptom-neural correlation analysis, reveals a quantitative relationship between this compensation and clinical symptoms. The findings indicate that repositioning therapy not only corrects the peripheral vestibular disorder but also fundamentally promotes the normalization of central sensory weight redistribution, thereby deepening our understanding of the neural mechanisms underlying visual dependence in BPPV.

### 4.4 Clinical symptom improvement and neural correlation

Clinically, significant post-repositioning reductions in DHI and VAS scores align with established evidence (46). DHI quantifies vertigo’s impact on daily lief (47), while VAS reflects subjective symptom intensity (48); their improvement validates the clinical efficacy of repositioning. This correlation suggests spatiotemporal coupling between neural adaptive remodeling and symptom resolution, positioning fNIRS as a clinically relevant tool with mechanistic explanatory power for decoding neurofunctional network dynamics in vestibular disorders.

### 4.5 Study significance and limitations

This study represents the first systematic application of fNIRS to characterize the neural compensation profile in patients with BPPV. By successfully capturing alterations in FC strength within brain networks following repositioning therapy, leveraging the high temporal resolution of fNIRS, this work fills a significant research gap by establishing a novel neuroimaging biomarker in this field.

Moreover, regarding clinical value, the study identified abnormally enhanced FC strength between the middle temporal gyrus and motor/somatosensory cortices, providing objective evidence supporting the spatial perception compensation mechanism and establishing a quantitative correlation between FC strength and clinical symptom severity. Methodologically, the study capitalized on the inherent high tolerance of fNIRS to motion artifacts, thus overcoming the limitations of traditional functional magnetic resonance imaging (fMRI) for investigating vertigo, while implementing a standardized experimental protocol to ensure data reliability.

However, this study has the following limitations: First, while the sample size of 50 BPPV patients is consistent with common standards for neuroimaging studies of vestibular disorders, significant differences exist between specific clinical subtypes—such as bilateral BPPV, ipsilateral multi-canal BPPV, and light cupula (LC-BPPV)—regarding repositioning techniques and neural compensation mechanisms; consequently, these specific subtypes were excluded to ensure the stability of neuroplasticity observations. Future research should expand the sample size and employ stratified enrollment to systematically include all clinical subtypes, enabling a systematic comparison of vestibular-cortical network reorganization across distinct pathological variants.

Second, the inherent spatial resolution and limited penetration depth of fNIRS constrain the localization accuracy for key deep brain structures; it is therefore recommended that subsequent studies adopt a multimodal integration approach, combining fNIRS with techniques such as electrophysiology and fMRI, as such multimodal methodologies hold promise for overcoming current limitations and advancing the development of precision medicine in vestibular rehabilitation. To accurately capture the immediate neural adjustments following repositioning treatment, the second fNIRS assessment was conducted 30 min after the procedure. This time point was selected to minimize interference from short-term discomfort and to elucidate the rapid initiation of central compensatory mechanisms; however, it does not account for the potentially longer time course of neuroplasticity, which may unfold over days or weeks. Full compensation and adaptation within the vestibular system involve a multi-stage process, ranging from short-term modulation to long-term structural reorganization. The findings of this study primarily reflect early, rapid changes in brain network activity after BPPV repositioning, which may be associated with the release of cognitive load. Future studies employing longitudinal designs with multiple time points-such as at 24 h, 7 days, and 1 month-would help delineate the complete dynamic trajectory from vestibular recovery to the maturation of central neuroplasticity, thereby providing deeper mechanistic insights.

## Data Availability

The raw data supporting the conclusions of this article will be made available by the authors, without undue reservation.
